# HoxA-11 and FOXO1A Cooperate to Regulate Decidual Prolactin Expression: Towards Inferring the Core Transcriptional Regulators of Decidual Genes

**DOI:** 10.1371/journal.pone.0006845

**Published:** 2009-09-03

**Authors:** Vincent J. Lynch, Kathryn Brayer, Birgit Gellersen, Günter P. Wagner

**Affiliations:** 1 Department of Ecology and Evolutionary Biology, Yale University, New Haven, Connecticut, United States of America; 2 Endokrinologikum Hamburg, Hamburg, Germany; Cincinnati Children's Research Foundation, United States of America

## Abstract

During the menstrual cycle, the ovarian steroid hormones estrogen and progesterone control a dramatic transcriptional reprogramming of endometrial stromal cells (ESCs) leading to a receptive state for blastocyst implantation and the establishment of pregnancy. A key marker gene of this decidualization process is the prolactin gene. Several transcriptional regulators have been identified that are essential for decidualization of ESCs, including the Hox genes HoxA-10 and HoxA-11, and the forkhead box gene FOXO1A. While previous studies have identified downstream target genes for HoxA-10 and FOXO1A, the role of HoxA-11 in decidualization has not been investigated. Here, we show that HoxA-11 is required for prolactin expression in decidualized ESC. While HoxA-11 alone is a repressor on the decidual prolactin promoter, it turns into an activator when combined with FOXO1A. Conversely, HoxA-10, which has been previously shown to associate with FOXO1A to upregulate decidual IGFBP-1 expression, is unable to upregulate PRL expression when co-expressed with FOXO1A. By co-immunoprecipitation and chromatin immunoprecipitation, we demonstrate physical association of HoxA-11 and FOXO1A, and binding of both factors to an enhancer region (−395 to −148 relative to the PRL transcriptional start site) of the decidual prolactin promoter. Because FOXO1A is induced upon decidualization, it serves to assemble a decidual-specific transcriptional complex including HoxA-11. These data highlight cooperativity between numerous transcription factors to upregulate PRL in differentiating ESC, and suggest that this core set of transcription factors physically and functionally interact to drive the expression of a gene battery upregulated in differentiated ESC. In addition, the functional non-equivalence of HoxA-11 and HoxA-10 with respect to PRL regulation suggests that these transcription factors regulate distinct sets of target genes during decidualization.

## Introduction

The successful establishment of pregnancy is dependent on proper growth and development of the uterine endometrium in preparation for blastocyst implantation. This complex process involves secretory transformation of glandular epithelial cells followed by decidualization of stromal cells [1]. The process of decidualization is poorly understood at the molecular level, but a key stimulus is progesterone acting on estrogen-primed endometrial stromal cells (ESCs) leading to dramatic transcriptional reprogramming [2]. Among the numerous genes that are upregulated during decidualization, prolactin (PRL) is one of the most dramatically induced, and decidua-derived PRL is one of the most abundant secretory products in the amniotic fluid [1], [3]–[6].

In the event of successful pregnancy, PRL expression in decidualized endometrial stromal cells persists until parturition [7]–[9]. Numerous functions are ascribed to decidual PRL including regulation of epithelial cell differentation, trophoblast growth, angiogenesis and modulation of the immune system [1], [4]. In rodents, an important role for decidual PRL is the suppression of genes detrimental to pregnancy, such as the cytokine interleukin-6 and the steroidogenic enzyme 20α-hydroxysteroid dehydrogenase [3], [10], [11]. Thus, the proper regulation of decidual PRL is essential for implantation and pregnancy.

In humans, decidual PRL is transcribed from an alternative promoter located upstream of the non-coding exon 1a, approximately 6 kb upstream of the promoter driving PRL expression in the pituitary from exon 1b [12]. Utilization of the alternative promoter ensures tissue-specific regulation of PRL transcription, while the protein encoded by the decidua- and the pituitary-specific transcripts is identical [12], [13]. The decidual PRL (dPRL) enhancer/promoter region is composed of two transposable elements in humans, MER20 and MER39 [13]. The eutherian-specific MER20 is located at −395/−148 relative to the transcriptional start site of the decidual transcript and contains binding sites for CCAAT/enhancer-binding protein β (C/EBPβ) [14], [15] and FOXO1A [14], [16], [17]. An essential ETS1 binding site [18], [19] and a CREB site [20], [21] is found in the primate-specific MER39 that also encodes exon 1a.

The MER20 enhancer region also includes several binding sites for abdominal-B (Abd-B) related Hox proteins (Hox paralog groups 9–13; this study). Expression of the Abd-B related HoxA genes HoxA-10 and HoxA-11 along the paramesonephric (Müllerian) duct is essential for the development and function of the female reproductive tract [22]. These genes continue to be expressed in the adult uterus and are required for successful blastocyst implantation in the mouse [23]–[26] suggesting that they play an active role in the regulation of decidual gene expression.

We previously observed a cooperative interaction of HoxA-11 with FOXO1A in regulating decidual PRL expression [27]. In this study we further characterize the ability of HoxA-11 to activate decidual PRL expression using siRNA-mediated knockdown, HoxA-11 overexpression and reporter gene assays. The results indicate that HoxA-11 is an intrinsic repressor of PRL expression but when combined with FOXO1A switches to a potent activator. Based on our own and published knockout, knockdown, functional and physical interaction data, we infer a decidual-specific enhanceosome that potentially regulates a battery of genes involved in endometrial differentiation such as PRL.

## Results

### Identification of transcription factor binding sites in the dPRL enhancer

**Figure 1:**
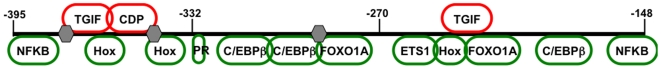
Structure of the decidual PRL (dPRL) enhancer. Schematic of transcription factor binding sites in the MER20 dPRL enhancer. The region −395 to −148 relative to the transcriptional start site, which corresponds to the MER20 transposon is shown. Binding sites for activators are shown in green and for repressors in red. Oct-1 sites are shown as gray hexagons.

Several transcription factors essential for dPRL expression have been identified, including FOXO1A which has functional binding sites in the dPRL enhancer [14]. Interestingly, FOXO1A has been shown to bind HoxA-10 [31], suggesting other Hox genes may play a direct role in regulating PRL expression and cooperation between FOXO1A and HoxA-11 has been observed to regulating dPRL expression [27], suggested a direct role for Hox genes in regulating dPRL expression. To identify potential Hox binding sites in the dPRL enhancer, we searched the human dPRL enhancer (chr6:22,411,225-22,411,422) with TRANSFAC for high-scoring transcription factor binding site matches (>0.90 core match identity). In addition to the previously known C/EBPβ, FOXO1A and ETS1 sites, three potential Hox binding sites were identified in the dPRL enhancer, as well as binding sites for nuclear factor KappaB (NFκB) subunit c-Rel, TG-interacting factor (TGIF), CAAT displacement protein (CDP) and the ubiquitous cofactor Oct-1 (Fig. 1).

### HoxA-11 is necessary, but not sufficient, for PRL expression

**Figure 2:**
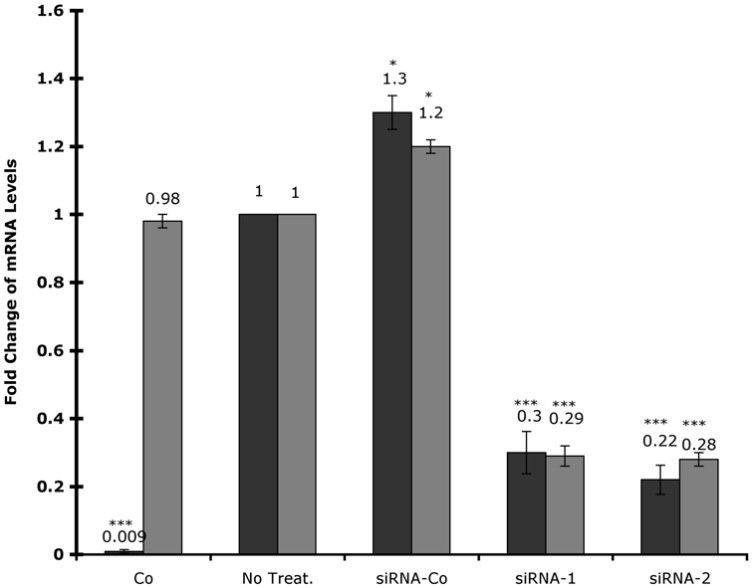
Effect of HoxA-11 knockdown on intrinsic HoxA-11 and PRL mRNA expression. The expression of PRL (dark gray bars) and HoxA-11 (light gray bars) was assayed in undifferentiated, mock-transfected ESC (UnDiff), or in differentiated cells (Diff) that had not been transfected (No Treat.) or had been transfected with random sequence siRNA control (siRNA-Co), or with two different siRNAs targeting HoxA-11 (siRNA-1, siRNA-2). Results are shown as fold changes relative to expression levels in differentiated control cells (set to 1). n = 6 replicate experiments, means±SEM. *, P<0.05; ***, P<0.001 (t-test compared to No Treat.).

To determine if HoxA-11 was necessary for induction of PRL expression, we transfected differentiating human endometrial stromal cells (hESC) with two siRNAs targeting different sites of the human HoxA-11 mRNA. In untransfected hESC, PRL expression was induced over 100-fold 48 hr after differentiation with cAMP analog and the progesterone analog medroxyprogesterone acetate (MPA), while the HoxA-11 mRNA level remained unchanged (Fig. 2). Transfection with HoxA-11 specific siRNAs markedly reduced both HoxA-11 (>70% reduction) and PRL (>70% reduction) expression in differentiated cells, while transfection with a random sequence control siRNA (siRNA-Co) led to a small enhancement of both HoxA-11 and PRL expression (20–30% increase). Further experiments indicated that treatment of hESC with transfection agents alone can induce both HoxA-11 and PRL expression (data not shown).

**Figure 3:**
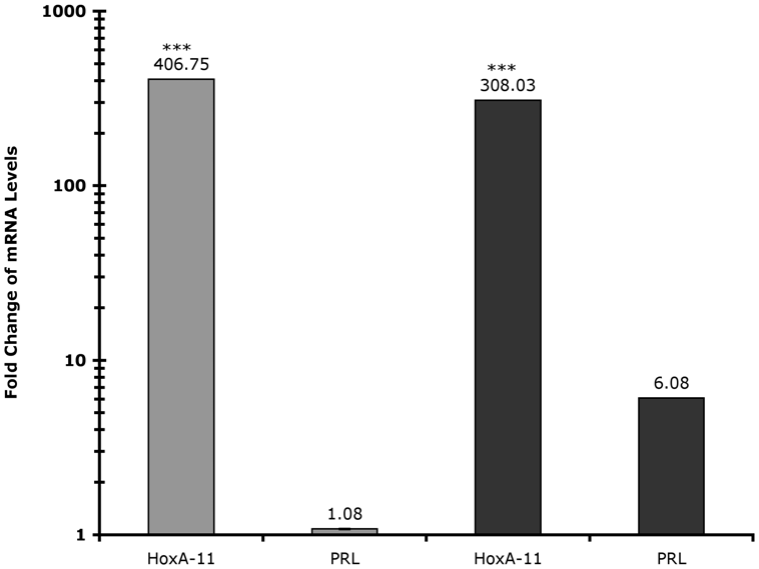
Effect of HoxA-11 overexpression on PRL expression. The expression of PRL mRNA was assayed after overexpression of HoxA-11 in undifferentiated (light gray bars) and differentiated (dark gray bars) ESC. Results are shown as fold changes relative to PRL expression in undifferentiated and differentiated mock-transfected cells, respectively. HoxA-11 expression was confirmed in undifferentiated (light gray bars) and differentiated (dark gray bars) ESC. HoxA-11 enhanced upregulation of PRL only in differentiated cells. Note, the y-axis (fold change) is log-scale. n = 6 replicate experiments, means±SEM. ***, P<0.001 (t-test, overexpression compared to undifferentiated and differentiated, respectively).

While the siRNA-mediated knockdown indicated that HoxA-11 is necessary for expression of PRL in decidual cells, most Hox genes exert their effects though associations with cofactors and transcription factors that impart activator or repressor functions [28], [29]. To identify if HoxA-11 alone is sufficient to induce PRL expression, we overexpressed HoxA-11 in undifferentiated and differentiated hESC and measured PRL expression. Overexpression of HoxA-11 had no effect on PRL expression in undifferentiated hESC, while overexpression of HoxA-11 in differentiated hESC led to a marked increase in PRL expression (6-fold) relative to PRL expression in differentiated hESC transfected with empty expression vector alone (Fig. 3). These results indicate that a hormone-inducible cofactor is required for HoxA-11 mediated activation of decidual PRL.

### HoxA-11 functionally interacts with FOXO1A

**Figure 4:**
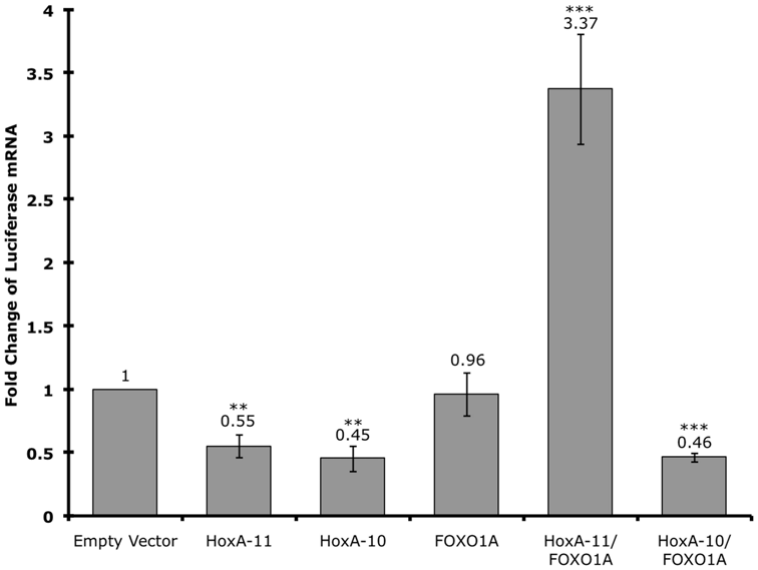
HoxA-11 and FOXO1A functionally cooperate to upregulate PRL expression. The expression of luciferase was assayed in HeLa cells transiently transfected with the dPRL-332/luc3 decidual PRL enhancer reporter construct, cotransfected with either empty expression vector, HoxA-11 alone, HoxA-10 alone, FOXO1A alone, or FOXO1A cotransfected with either HoxA-11 or HoxA-10. Results are shown as fold changes relative to luciferase expression in cells cotransfected with dPRL-332/luc3 and empty expression vector. n = 8 replicate experiments, means±SEM. **, P<0.05; ***, P<0.001 (t-test compared to empty vector).

Examination of the decidual PRL enhancer identified several potential Hox binding sites, including one immediately adjacent to FOXO1A and ETS1 binding sites (Fig. 1). Furthermore, FOXO1A is upregulated both *in vivo* and *in vitro* upon decidualization [6], [14], suggesting that HoxA-11 and FOXO1A may interact to regulate decidual PRL expression. To test if there was a functional association of HoxA-11 and FOXO1A we cotransfected HeLa cells with a luciferase reporter construct containing the dPRL enhancer (dPRL-332/luc3) and expression vectors for HoxA-11, FOXO1A or both (Fig. 4). Transfection of HoxA-11 alone repressed expression of luciferase from the decidual PRL enhancer, consistent with the finding that HoxA-11 generally functions as a repressor [32]. Transfection of FOXO1A alone had no effect on luciferase expression, while transfection of both HoxA-11 and FOXO1A lead to over 3-fold activation from the PRL enhancer (Fig. 4). These data suggest that HoxA-11 and FOXO1A act cooperatively to upregulate PRL from the decidual PRL enhancer, and that association with FOXO1A switches HoxA-11 from a repressor to an activator.

### HoxA-11 and HoxA-10 are not functionally equivalent regulators of PRL expression

Previous studies have shown that HoxA-10 functionally and physically interacts with FOXO1A to regulate decidual IGFBP-1 expression [30], [31]. However, gene expression profiling in peri-implantation mouse uterus did not identify PRL as a HoxA-10 target gene. These results suggest that although both HoxA-11 and HoxA-10 can physically and functionally associate with FOXO1A, their regulatory effects are target gene specific. To test if HoxA-10 can regulate expression from the decidual PRL enhancer in a similar way as HoxA-11, we cotransfected HeLa cells with the dPRL-332/luc3 luciferase reporter construct and expression vectors for HoxA-10, and both HoxA-10 and FOXO1A (Fig. 4). Like HoxA-11, HoxA-10 alone represses expression from the dPRL-332/luc3 luciferase reporter construct. Remarkably, however, while co-expression of HoxA-11 and FOXO1A upregulates expression from the dPRL enhancer, co-expression of HoxA-10 and FOXO1A failed to upregulate expression from the dPRL enhancer. These results indicate that these Hox genes are functionally non-equivalent with respect to their ability to regulate decidual PRL expression, even though they are able to associate with similar cofactors.

### Identification of HoxA-11/FOXO1A binding sites

**Figure 5:**
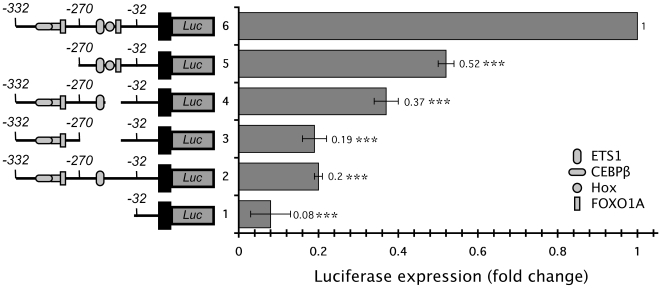
Identification of the HoxA-11/FOXO1A responsive region of the dPRL promoter. The expression of luciferase was assayed in HeLa cells transiently transfected with HoxA-11 and FOXO1A and (from top to bottom) either dPRL-332/luc3, dPRL-270/luc3, dPRL(−332/−232)/−32/luc3, dPRL(−332/−270)/−32/luc3, dPRL-32/luc3 or dPRL-332/luc3mutHox. Results are shown as fold changes relative to luciferase expression in cells cotransfected with HoxA-11/FOXO1A and dPRL-332/luc3. A schematic of the location of transcription factor binding sites and deletions is shown. n = 6 replicate experiments, means±SEM. ***, P<0.001 (t-test compared to dPRL-332/luc3).

To identify the Hox and FOXO responsive region in the dPRL enhancer, we cotransfected HeLa cells with luciferase reporter constructs containing deletion mutants of the dPRL enhancer (dPRL-332/luc3, dPRL-270/luc3, dPRL(−332/−232)/−32/luc3, and dPRL(−332/−270)/−32/luc3) and expression vectors for HoxA-11 and FOXO1A (Fig. 5). Deletion of the previously characterized cluster of two C/EBP and a FOXO site (reporter construct dPRL-270/luc3) reduced luciferase expression by ∼50% (Fig. 5), reflecting the previously reported cooperativity between FOXO1A and C/EBP [13]. Deletion of the adjacent Hox/FOXO sites (reporter construct dPRL(−332/−232)/−32/luc3) reduced luciferase expression by ∼60% and deletion of the entire ETS/Hox/FOXO binding site complex (construct dPRL(−332/−270)/−32/luc3) reduced luciferase expression by ∼80% of the full-length dPRL enhancer (Fig. 5). Similarly, mutating the Hox site (TTAT→GCGC) reduced luciferase expression by ∼80% of the full-length dPRL enhancer (Fig. 5).

### HoxA-11 and FOXO1A physically interact

**Figure 6:**
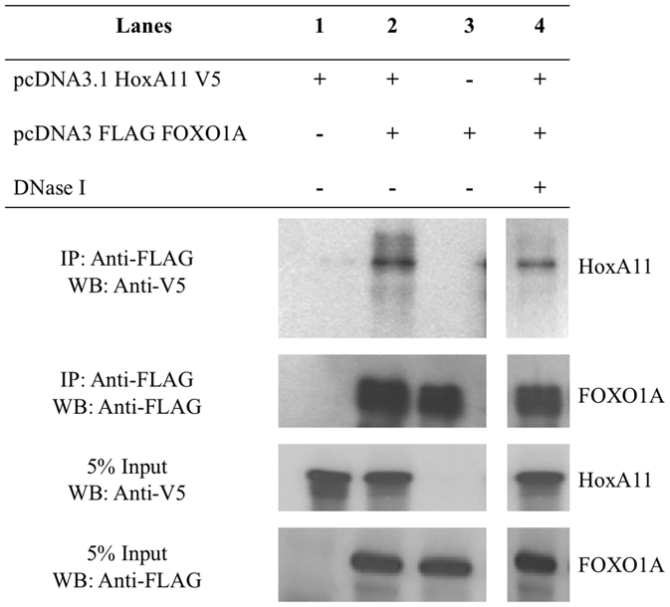
Physical interaction between HoxA-11 and FOXO1A. HoxA-11-V5/His and Flag-FOXO1A were overexpressed in HeLa cells and subjected to co-immunoprecipitation with anti-FLAG agarose beads. Eluates, without or with DNase treatment, were analyzed by Western blotting with V5 antibody, followed by re-probing with FLAG antibody.

The functional association between HoxA-11 and FOXO1A and their adjacent binding sites suggests they physically interact. This was investigated by in vivo co-immunoprecipitation assays. HeLa cells were transfected with a HoxA11-V5/His fusion protein and either empty pcDNA3.1(+) vector or Flag-FOXO1A pcDNA3.1. Immunoprecipitation was performed with anti-FLAG agarose beads and assayed by serial western blot using anti-V5-HRP and anti-Flag-HRP antibodies. HoxA11-V5/His was immunoprecipitated in the presence of Flag-FOXO1A (Fig. 6), but not controls indicating direct interaction between HoxA-11 and FOXO1A. Moreover, addition of DNase did not disrupt the interaction, indicating that the physical interaction between HoxA-11 and FOXO1A is not DNA-dependent (Fig. 6).

### Chromatin Immunoprecipitation (ChIP)

**Figure 7:**
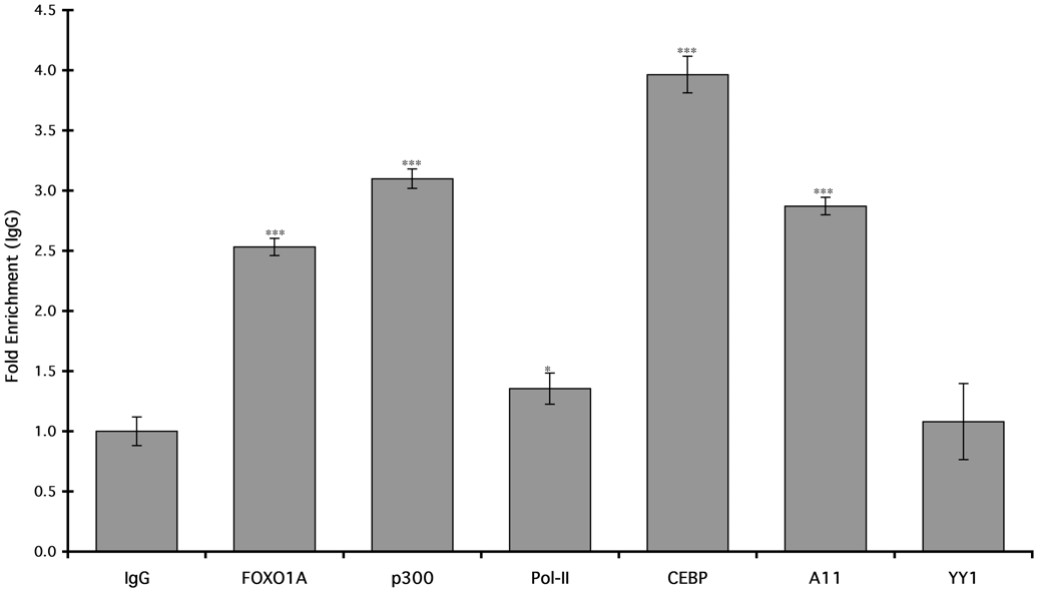
Chromatin immunoprecipitation assay for transcription factors at the dPRL enhancer. Chromatin immunoprecipiation was performed in differentiated hESC with either a control IgG, or antibodies to FOXO1A, p300, Pol-II, C/EBPβ, HoxA-11 or YY1. Results are shown as fold enrichment over IgG (after normalization to inputs). n = 6 replicate experiments, means±SEM. *, P<0.05; ***, P<0.001 (t-test compared to IgG).

To test if FOXO1A and HoxA-11 bind the dPRL enhancer *in vivo*, we performed chromatin immunoprecipitation (ChIP) on differentiated hESC followed by quantitative PCR to assay for the PRL MER20 with primers designed to amplify the region from −212 to −270. We found significant enrichment of HoxA-11 and FOXO1A on the dPRL enhancer in differentiated hESC compared to controls precipitated with control IgG (Fig. 7). Similarly, there was significant enrichment for C/EBPβ, which has been previously shown to bind the dPRL enhancer, and p300, a cofactor known to bind numerous enhancers. There was only minor but still significant enrichment for Pol-II, and no enrichment for YY1 (Fig. 7).

## Discussion

Hox proteins are transcription factors that play important roles in embryonic development and adult function of many tissues and organ systems. In the female reproductive tract, the Abd-B related Hox genes (Hox9-Hox13) are expressed along the developing paramesonephric (Müllerian) duct and continue to be expressed in adults. While many Hox genes are functionally redundant between paralog group members, the Adb-B related HoxC and HoxD genes regulate endometrial proliferation and HoxA genes regulate endometrial differentiation and are required for successful blastocyst implantation [23], [24], suggesting the distinct roles of these transcription factors is mediated by differential activation of downstream target genes. Thus, identification of Hox target genes is important for understanding their roles in endometrial function; however, only three direct target genes of HoxA-10, namely IGFBP1 [30], [31], ITB3 [33] and EMX2 [34], and thus far no targets of HoxA-11 have been identified in the endometrium.

By siRNA-mediated knockdown, overexpression, and deletion analysis of the dPRL enhancer, we identified HoxA-11 as a direct regulator of PRL expression. This adds HoxA-11 to the list of transcription factors that are directly involved in dPRL activation, such as AP-1 [35], C/EBPβ [14], [15], ETS1 [18], [19], FOXO1A [14], [16], [17] and PGR [36], [37]. These results indicate that PRL expression in the endometrium is a tightly regulated process that is dependent on the coordinated action of multiple transcription factors, which likely acts to canalize decidualization stimuli and ensure inappropriate differentiation does not result from low level or erroneous stimulation of stromal cells.

ChIP analysis of the dPRL enhancer in differentiated stromal cells demonstrated that HoxA-11 and FOXO1A associate with the dPRL enhancer, likely as part of an enhancer complex that includes p300, C/EBPβ and possibly Pol-II. Interestingly, previous work has demonstrated that a composite C/EBPβ/FOXO1A site upstream of the HoxA-11/FOXO1A site binds C/EBPβ and FOXO1A, which recruit PGR to the enhancer [36], [37]. These results suggest that proteins bound to the C/EBPβ/FOXO1A and HoxA-11/FOXO1A composite sites may directly interact through DNA-bending or looping, or that other cofactors bridge these two protein complexes.

Several of the transcription factors with conserved binding-sites in the decidual PRL enhancer have been characterized with respect to endometrial function through knockout and knockdown studies. For example, female HoxA-11 knockout mice are infertile with specific defects in blastocyst attachment and implantation [23], [24], and knockout of C/EBPβ impairs decidual differentiation [38]. In human ESC, knockdown of ETS1 significantly reduces PRL expression [18], [19] and FOXO1A [16], [17]. C/EBPβ [14], [15], FOXO1A [14] and AP-1 [35] have also been demonstrated to bind sites within the enhancer and are required for dPRL expression.

Our work highlights the role of FOXO1A, a transcription factor up-regulated in decidualization, to switch the constitutively expressed repressor HoxA-11 to a potent co-activator. Furthermore, HoxA-10, which can also interact with FOXO1A is not able to upregulate PRL expression. Together these data further underpin the concept that multiple, but specific, transcription factors function cooperatively to upregulate PRL in differentiating ESC, and suggest that this core set of transcription factors physically and functionally interact to drive the expression of a gene battery upregulated in differentiated ESC. Remarkably, there are over 20,000 MER20s in the human genome and many located near genes expressed in differentiated stromal cells (unpublished observations: V.J. Lynch, R. Leclerc, and G.P. Wagner). If transcription factor binding is a general feature of MER20s, particularly by FOXO1A, HoxA-11, CEBPβ and p300, then MER20 derived regulatory elements may be orchestrators of ESC differentiation.

## Methods

### Identification of transcription factor binding sites in the dPRL enhancer

Potential transcription factor binding sites in the human MER20-derived decidual PRL enhancer (dPRL) were identified using the MATCH program (http://.gene-regulation.com/) utilizing TRANSFAC binding site matrices with a match cut-off selected to minimize the sum of false positive and false negative results. Only binding site matches with >90% identity to the core binding site motif are reported here.

### Reporter constructs and expression vectors

Generation of the dPRL promoter/luciferase reporter constructs in the pGL3-Basic plasmid (Promega) has been described [12]. Briefly, construct dPRL-332/luc3 contains the wild type dPRL promoter sequence −332 to +65 relative to the human dPRL transcriptional start site [12]. Similarly, the dPRL-270/luc3 construct contains −270 to +65 relative to the human dPRL transcriptional start site, and dPRL-32/luc3 encompasses the minimal dPRL promoter element (−32 to +65). The internal deletion constructs dPRL(−332/−270)/−32/luc3 and dPRL(−332/−232)/−32/luc3 were generated by placing the elements −332/−270 or −332/−232, respectively, immediately upstream of the minimal dPRL promoter element in dPRL-32/luc3. The dPRL-332luc3mutHox construct mutated the Hox binding site spanning −232 to −236 from TTAT to GCGC using the QuickChange site directed mutagenesis kit (Stratagene, Texas USA).

The full-length human HoxA-11 and HoxA-10 cDNAs were cloned into the pcDNA3.1/V5-His Topo TA expression vector (Invitrogen) to create HoxA11-V5/His and HoxA10-V5/His fusion proteins, respectively. N-terminal Flag tagged human FOXO1A was purchased from Addgene (Addgene plasmid 13507).

### Cell culture and transient transfection

Human endometrial stromal cells immortalized with telomerase (ATCC, Cat. No. CRL-4003) and HeLa cells were grown in steroid-depleted DMEM, supplemented with 5% charcoal-stripped calf-serum and 1% antibiotic/antimycotic (ABAM). For induction of decidualization, ESC were treated with 0.5 mM 8-Br-cAMP (Sigma) and 1 µM medroxyprogesterone acetate. At 80% confluency, cells were transfected using TransIT-LT1 (Mirus, Cat. No. MIR2304) according to the manufacturer's protocol to introduce the following vectors: for RNAi mediated silencing, either of two HuSH 29mer shRNA plasmids expressing siRNA directed against two different target sequences of human HoxA-11 mRNA (Origene, Cat. No. 312366) were employed; vehicle control samples were transfected with a HuSH 29mer shRNA plasmid expressing a non-targeting random sequence siRNA. For overexpression and reporter gene studies, cells received expression vectors for human HoxA-11, HoxA-10, FOXO1A and the decidual PRL enhancer/promoter reporter constructs. Cells were harvested and RNA collected 3 days after differentiation/transfection.

Real-time PCR reagents, including TaqMan FAST Universal PCR Master Mix, and HoxA-11, PRL and GADPH endogenous control primer/probe sets were purchased from Applied Biosystems. Luciferase expression was measured using a TaqMan Primer/Probe set designed to the luciferase gene in pGL3-Basic reporter vector.

### Co-Immunoprecipitation Assay

HeLa cells were cultured in Dulbecco's modification of Eagle's medium (DMEM; Cellgro) with 4.5 g/L glucose and L-glutamine and without sodium pyruvate, plus 5% fetal bovine serum (Gibco) and 1x ABAM (Gibco). Prior to transfection, cells were plated at a density of ∼4.4×10^6^ cells/mL on 10 cm plates. The next day, Hox-A11-V5/His construct was transfected into HeLa cells with either empty pcDNA3.1(+) vector, or Flag-Foxo1A pcDNA3.1 vector using Lipofectamine 2000 (Invitrogen) per the manufacturer's protocol. A total of 12 µg of DNA was mixed with Lipofectamine (1∶3) in 3 mL OptiMEM reduced serum media (Gibco) and incubated for 4 hours at 37°C, before addition of 7 mL DMEM. After 16 hours the transfection media was removed and replaced with fresh DMEM, and the cells were incubated an additional 24 hours before harvesting.

After removing DMEM and washing cells twice with PBS, 1 mL ice-cold lysis buffer (20 mM Tris, pH 8.0, 40 mM KCl, 10 mM MgCl_2_, 10% glycerol, 1% Triton X-100, 1x Complete EDTA-free protease inhibitor cocktail (Roche), 1x PhosSTOP (Roche)) was added to each plate and cells were harvested by scraping with a rubber spatula. Harvested cells were transferred to borosilicate glass tubes, and incubated on ice for 10 minutes prior to the addition of 5 M NaCl to a final concentration of 420 mM. Cells were sonicated in an ultrasonic water bath (Sonicor) for 2×15 seconds, followed by an additional incubation on ice for 60 minutes, and another round of sonication. Whole cell lysate was cleared by centrifugation at 10,000 rpm for 30 minutes at 4°C, and supernatant was transferred to a clean microfuge tube. Total protein concentration was determined by Bradford analysis. After equilibrating protein concentrations, 1 mL of sample was mixed with 40 µL of M2 anti-FLAG agarose beads (Sigma) pre-washed with TNT buffer (50 mM Tris-HCl, pH 7.5, 150 mM NaCl, 0.05% Triton X-100), and rotated overnight at 4°C.

The following day, samples were treated with 50 U DNase (Roche) and 2.5 µg RNase (Roche) for 60 minutes at room temperature, as indicated. Samples were washed 3x with 1 mL of Hepes Wash (10 mM Hepes, 150 mM NaCl, 0.5% Triton X-100). Between each wash, agarose beads were collected by centrifugation at 1000 rpm for 1 min at 4°C and supernatant was removed. After the final wash, beads were re-suspended in elution buffer (500 mM Tris pH 7.5, 1 M NaCl), and incubated for 1 hour at 4°C. Agarose beads were pelleted at 5000 rpm for 1 min, and supernatant was transferred to a clean tube containing loading buffer (50 mM Tris pH 6.8, 100 nM DTT, 2% SDS, 0.1% bromophenol blue, 10% glycerol) and resolved on a 12% SDS-PAGE gel at 100 V. Proteins were transferred to a PVDF membrane (100 V for 1.5 hours). After transfer, membrane was blocked with TBS Tween20+BSA (25 mM Tris, 150 mM NaCl, 3% BSA), then incubated with anti-V5-HRP antibody (1∶5000; invitrogen) for 2 hours. Bands were visualized using the ECL SuperSignal West Pico Chemiluminescent Substrate (Thermo Scientific). Membranes were stripped with Restore Western Blot stripping buffer (Thermo Scientific) and re-probed with anti-Flag-HRP antibody (1∶100,000; Sigma).

### Chromatin Immunoprecipitation

For chromatin immunoprecipitation (ChIP), the EZ-Zyme Chromatin Prep kit (Upstate Millipore, Billerica, MA) was used following the manufacturer's protocol. Briefly, crosslinking was done on differentiated hESC with 1% formaldehyde, followed by DNA fragmentation. The equivalent of 10^6^ cells was used for each immunoprecipitation. The nuclear lysate was precleared with protein G magnetic beads and incubated over night at 4°C with protein G magnetic beads, and 2 µg of either the antibodies against FOXO1A, p300, Pol-II, CEBPβ, HoxA-11 and Y11, or with the rabbit IgG as negative control (all from Santa Cruz Biotechnology, Santa Cruz, CA). Enrichment of the dPRL enhancer was evaluated by qPCR using 1/50 of the immunoprecipitated chromatin as template and the Power SYBR Green PCR Master Mix (Applied Biosystems, Foster City, CA).
